# The Expanding Role of Probiotics in Human Health

**DOI:** 10.3390/nu17071116

**Published:** 2025-03-24

**Authors:** George Stavrou, Katerina Kotzampassi

**Affiliations:** 1 1st Surgical Department, 417 NIMTS (Army Share Fund) Hospital, 11521 Athens, Greece; 2 Department of Surgery, Aristotle University of Thessaloniki, 54636 Thessaloniki, Greece

Since the 1990s, it has been widely documented that probiotics, either alone or in combination with prebiotic supplements, play a vital role in host health and disease management. However, at present, with increased knowledge of their specific actions, i.e., antioxidant and immunomodulatory actions and enhancement of epithelial barrier function—most likely driven through the modulation of the local microbiota—and their beneficial effects in disease prevention and treatment, a significant proportion of experimental research, in addition to hundreds of clinical applications, is currently focusing on their use [1,2].

The word “probiotics” tends to be automatically linked to the gut microbiota; however, almost all scientists today recognize that the gut, despite being the oldest recognized and perhaps even central “host” organ, is not unique [1]; other sites, including the oral cavity, the lungs, the urinary tract/bladder, and the skin microbiota, are also recognized and crucially implicated both in local and remote-organ disease [3,4,5,6]. Postbiotics, recently defined as “inanimate microorganisms and/or their components that confer a health benefit on the host”, were used as cosmetics for the enhancement of skin health long before they were renamed postbiotics [7]. Moreover, they have traditionally been used in immunocompromised patients rather than as live probiotics, eliminating the theoretical risk of bacteremia induction while also exerting equally positive effects. Prebiotics, being the physical background for bacterial growth, were defined 30 years ago as “non-digestible food ingredients that beneficially affect the host, by selectively stimulating the growth and/or activity of one or a limited number of bacteria already resident in the colon” [2,8,9].

The growing belief that diseases of any type and body system—from atopic dermatitis to neurodegenerative disorders—are related to the destruction of the related microbiome has led to the hypothesis that, by modulating the local microbiome through a probiotic formula, disease can be prevented or even cured. Thus, the human microbiome has emerged as a central focus in medical research, revealing intricate connections between the composition of the microbiota (and not exclusively of the gut) and systemic health. The symbiotic relationship between the microbiota and the host affects metabolic functions, immune responses, and neurological processes through the gut–brain axis and even reproductive and dermatological health.

With growing evidence supporting the impact of beneficial bacteria, alone or with prebiotics, or alternatively postbiotics, on specific mechanisms of human biology and the two-way communication of local microbiomes via the nervous system, there is increasing interest in harnessing these bacterial elements both to prevent and treat illness and sustain and improve health.

The exceptional collection of both research articles and reviews in this Special Issue delves into the diverse applications of microbiome-modulating interventions, providing insights into their physiological and clinical implications.

One of the key areas of investigation in this issue is metabolic health and chronic disease management. Liu et al. [10] present a meta-analysis of *Akkermansia muciniphila*, a new-generation probiotic, known for its beneficial effects in preventing the onset of type-2 diabetes and obesity through the promotion of the gut production of short-chain fatty acids—thus improving insulin resistance and the host metabolism—along with the stimulation of beneficial gut bacteria, such as *Bifidobacteria*. The authors emphasize that *A. muciniphila* treatment significantly reduces fasting blood glucose levels, by improving glucose tolerance and increasing blood insulin levels, thus being effective in preventing type 2 diabetes in animals, while significantly reducing the increase in body weight by 10.4%. Jang et al. [11] contribute to this argument with their study on *Lactobacillus delbrueckii* subsp. lactis CKDB001, aimed at demonstrating, in a murine model of high-fat diet-induced obesity, its capacity to counteract metabolic disturbances. The study results highlight improvements in insulin sensitivity, reductions in serum triglycerides and hepatic lipid accumulation, and the alleviation of metabolic dysfunction-associated steatotic liver disease. In the same manner, Zhang et al. [12], in a clinical trial, examine the beneficial effects of *Weizmannia coagulans* BC99 in long-term alcohol consumers. Patients randomized to a *W. coagulans* BC99 group for 60 days demonstrated, compared to the placebo, improvements in hepatic enzyme profiles and anti-inflammatory factors, in addition to an increased abundance of *Muribaculaceae*, considered the key factor in alleviating alcohol-induced liver damage in long-term alcohol consumers. The potential of *W. coagulans* to promote gut–liver axis homeostasis could have further implications for managing hepatic disorders in general.

Expanding beyond metabolic conditions, López-Yerena et al. [13] review papers on the cardiovascular benefits exerted by probiotics, emphasizing the potential mechanisms through which specific strains may lower cholesterol, reduce inflammation, and improve endothelial function. Their findings focus on the impact of probiotics on major cardiovascular risk factors, such as hypertension, type 2 diabetes mellitus, metabolic syndrome, and hypercholesterolemia, and thus their use for the secondary prevention of coronary artery disease, acknowledging them as adjunct “therapies” in cardiovascular disease prevention. However, further interpretation of their properties, in terms of the optimal strains, dosage, and treatment duration, in addition to the influence of genus and the delivery vehicle, is required.

The role of probiotics in immune function and gastrointestinal health is another important topic. Wada et al. [14], in their innovative clinical study, investigate the immunomodulatory properties of *Lactobacillus helveticus* GCL1815, which optimally prevents the onset of systemic and local common cold symptoms in healthy adults. Their findings clearly indicate that *L. helveticus* GCL1815 enhances the response to viruses by activating two distinct dendritic cell pathways: significantly increasing the expression of [i] CD86 and HLA-DR in plasmacytoid dendritic cells from the fourth week of intake and [ii] the expression of HLA-DR in type 1 conventional dendritic cells at 8 weeks of intake, while also maintaining stable levels of CD86 in type 1 conventional dendritic cells, which otherwise decrease in the placebo. In the same vein, Naghibi et al. [15], in a randomized, double-blind controlled trial, evaluate the effects of the postbiotic *Bifidobacterium longum* CECT 7347 in healthy adults with mild-to-moderate gastrointestinal symptoms. Although minimal improvement was observed in gastrointestinal symptoms per se and visceral sensitivity tests, 8 weeks of treatment with this probiotic seems to promote gut health by increasing the abundance of butyrate-producing bacteria (mainly *Faecalibacterium* and *Anaerobutyricum*) while maintaining fecal calprotectin levels and reducing serum cholesterol levels. Additionally, Matar et al. [16] provide a comprehensive review of the intestinal barrier and its permeability by analyzing the harmful effects of specific food ingredients, the impact of altered permeability in pre-clinical models and human diseases, and the role of the microbiome and epigenomics in controlling epithelial barrier function. The authors then focus on the possibility of restoring gut integrity using beneficial microbiota interventions, emphasizing the interplay between probiotics, together with their active metabolites, and other secreted compounds, and the tight junction proteins in intestinal epithelial cells, which are crucial for preventing systemic inflammation and metabolic endotoxemia.

Stachelska et al. [17] analyze the health-promoting properties of probiotic bacteria linked to fermented milk beverages, emphasizing their role in preventing lifestyle-related diseases. In their study, the authors detail the modifications occurring in the intestinal microbiota that lead—through various mechanisms—to the strengthening of the immune system and the prevention of the diseases of modern life, i.e., obesity, diabetes, and possibly cancer. However, all of these conditions/diseases seem to be closely linked to one vicious cycle or another, influenced by independent parameters. This new direction in personalized medicine is gaining exceptional traction in probiota research as well. These results evidence the importance of screening across multiple age groups separately to provide details on how therapeutics impact the microbiome and, consequently, human health. Firrman et al. [18] highlight the influence of age in shaping the gut microbiome, with the dominant taxa together with their metabolites changing over a person’s lifetime, making it important to screen individuals in each age group to determine how therapeutics—in the present case, the human milk oligosaccharide 2′-fucosyllactose—as above, impact the microbiome and, consequently, human health.

Beyond the traditional domains of gut and metabolic health, this Special Issue also explores the expanding applications of beneficial bacteria research in reproductive medicine. Raimundo et al. [19] present a retrospective analysis of *Ligilactobacillus salivarius* PS11610, assessing its potential role as a one-month oral pretreatment in enhancing in-vitro fertilization success rates, particularly in the frozen embryo transfer group. Although the quality of embryos was lower than in the placebo group, there was an increase in the number of live births and biochemical pregnancy rates. However, further research in this field is required, including the possibility of topical application.

Lastly, in the field of dermatology/cosmetology science, Park et al. [20] examine the protective effects of *Limosilactobacillus fermentum* MG5368 and *Lactiplantibacillus plantarum* MG989 in UVB-induced skin damage. Their experimental study, conducted on both HaCaT cells and hairless mice, provides evidence that these two probiotic strains have the potential to be utilized as probiotic-based skin anti-photoaging formulations, since they can equally contribute to the promotion of collagen synthesis and hyaluronic acid restoration and thus maintain skin elasticity while inhibiting wrinkle formation.

To comply with regulation for the characterization of a microorganism as a specific probiotic strain, the pro- or postbiotics incorporated into a cosmetic regime should be both genetically and phenotypically defined. Based on this assumption, Theodorou et al. [21] analyze 14 published clinical trials and discuss the beneficial effects of those cosmeceuticals—fulfilling the above criteria—applied to volunteers with healthy skin. Their analysis emphasizes the growing consumer interest in microbiome-based skincare products and highlights the trend toward personalized skin care, which may lead to genomic sequencing and metabolomics to tailor probiotic and postbiotic treatments to individual skin microbiomes.

Collectively, these studies illustrate the growing recognition of the microbiome as a fundamental determinant of human health. As research continues to unravel the complex interactions between beneficial bacteria and host physiology—either as a generalized interface, as with the gut–brain axis, or at a local level, as with the skin microbiome—in health and disease, the field of medicine in the future will increasingly integrate microbiome-targeted strategies. It is our hope that this Special Issue will serve as a valuable resource, inspiring further exploration into the transformative potential of the human microbiome(s) in both preventive and therapeutic contexts.
